# FTZ Ameliorates Diabetic Cardiomyopathy by Inhibiting Inflammation and Cardiac Fibrosis in the Streptozotocin-Induced Model

**DOI:** 10.1155/2021/5582567

**Published:** 2021-09-28

**Authors:** Lexun Wang, Huijuan Wu, Yanyue Deng, Shengxi Zhang, Quxing Wei, Qianqian Yang, Shenghua Piao, Weijian Bei, Xianglu Rong, Jiao Guo

**Affiliations:** Guangdong Metabolic Diseases Research Center of Integrated Chinese and Western Medicine, Key Laboratory of Glycolipid Metabolic Disorder, Ministry of Education of China, Guangdong TCM Key Laboratory for Metabolic Diseases, Institute of Chinese Medicine, Guangdong Pharmaceutical University, Guangzhou, China

## Abstract

**Background:**

The pathogenesis and clinical features of diabetic cardiomyopathy (DCM) have been well studied in the past decade; however, effective approaches to prevent and treat this disease are limited. Fufang Zhenzhu Tiaozhi (FTZ) formula, a traditional Chinese prescription, is habitually used to treat dyslipidemia and diabetes. Recently, several studies have reported the therapeutic effects of FTZ on cardiovascular diseases. However, the effects of FTZ on DCM have not yet been fully elucidated. This study investigated the effects of FTZ on DCM and determined the mechanisms underlying its efficacy.

**Methods:**

Diabetes was induced in mice by intraperitoneal injection of streptozotocin; the mice were randomly divided into a control group (Con), diabetes group (DCM), and diabetes-treated with FTZ (DCM + FTZ). Myocardial structural alterations, fibrosis biomarkers, and inflammation were observed. Besides, the potential targets and their related signaling pathways were analyzed using network pharmacology and further verified by Western blot.

**Results:**

Diabetic mice showed significant body weight loss, hyperglycemia, and excessive collagen content in the cardiac tissue, while serum and myocardial inflammatory factors significantly increased. Nerveless, treatment with FTZ for 1 month significantly improved body weight, attenuated hyperglycemia, and alleviated diabetes-associated myocardial structure and function abnormalities. Furthermore, the serum levels of interleukin 12 (IL-12) and chemokine (C–C motif) ligand 2 (CCL2) as well as the mRNA levels of cardiac *IL-12*, *IL-6*, and C–C motif chemokine receptor 2 (*Ccr2*) reduced after FTZ treatment. Additionally, a total of 67 active compounds and 76 potential targets related to DCM were analyzed. Pathway and functional enrichment analyses showed that FTZ mainly regulates inflammation-related pathways, including MAPK and PI3K-AKT signaling pathways. Further investigation revealed that the activities of STAT3, AKT, and ERK were augmented in diabetic hearts but decreased in FTZ-treated cardiac tissues.

**Conclusion:**

Our results suggest that FTZ exhibits therapeutic properties against DCM by ameliorating hyperglycemia-induced inflammation and fibrosis via at least partial inhibition of AKT, ERK, and STAT3 signaling pathways.

## 1. Introduction

Diabetic cardiomyopathy (DCM), characterized by adverse structural remodeling, early onset diastolic dysfunction, and late-onset systolic dysfunction, is observed in diabetic patients that occurs in the absence of coronary artery disease, hypertension, valvular and congenital heart disease, leading to heart failure and death [1, 2]. Reportedly, DCM affects approximately 12% of diabetes patients, of whom approximately 22% are over 64 years old [3, 4]. With the increasing prevalence of diabetes, the harm caused by DCM will further expand, which has provoked research on the mechanisms underlying DCM. In recent years, a wide variety of mechanisms have been reported to be involved in DCM, including hyperglycemia, insulin resistance, cardiac fibrosis, inflammation, oxidative stress, and activation of cell death pathways [1, 5–7]. Moreover, inflammation, induced by persistent hyperglycemia, plays a key role in the development of DCM [1, 5, 7, 8]. In the diabetic heart, cytokines and chemokines secreted by infiltrative proinflammatory macrophages and lymphocytes contribute to the development of cardiomyocyte hypertrophy and a progressive fibrotic response, resulting in extracellular matrix (ECM) accumulation and fibrosis [1]. Although the pathogenesis of DCM has been extensively studied, efficient therapies are not available. There are several possible reasons for this. (1) Most mechanistic studies on DCM have been conducted in animals rather than humans [6]. These mechanisms in animal models may not be the same in humans. (2) Conventional medications for the treatment of diabetes in clinical practice are rarely used to treat DCM because they have no beneficial effects on heart function (such as glucagon-like peptide 1 receptor (GLP-1R) agonists and dipeptidyl peptidase-4 (DDP-4) inhibitors) or their potential to cause harmful cardiac involvement (such as metformin, which can cause lactic acidosis and thus aggravate heart damage, and thiazolidinediones, which can cause edema and heart failure (HF)) [8]. Thus, the clinical verification of traditional drugs and the secondary development of existing drugs are important in the prevention and treatment of DCM.

Fufang Zhenzhu Tiaozhi (FTZ) formula is an effective traditional Chinese prescription established by Professor Jiao Guo under the guidance of her new theory “Tiao Gan Qi Shu Hua Zhuo” for treating glycolipid metabolic disorders, consisting of *Ligustri Lucidi Fructus, Coptidis Rhizoma, Cirsii Japonici Herba, Salviae Miltiorrhizae Radix et Rhizoma, Eucommiae Cortex, Notoginseng Radix et Rhizoma, Atractylodes Macrocephala Rhizoma,* and *Citri Sarcodactylis Fructus* [9–13]. Our serial studies have identified the main components and potential targets of FTZ and demonstrated its protective effects in treating hyperlipidemia, diabetes, nonalcoholic fatty liver disease (NAFLD), and aging-induced osteoporosis through regulation of HMG-CoA reductase (HMGCR) and cholesterol 7-alpha hydroxylase (CYP7A1), attenuating insulin resistance, inhibiting the formation and activation of the nucleotide-binding oligomerization domain-like receptor protein 3 (NLRP3) inflammasome, and modulating sphingolipid, glycerophospholipid, and amino acid metabolisms, respectively [11, 14–16]. With the ability to alleviate inflammation and improve vascular endothelium function, FTZ is a promising drug for the treatment of cardiovascular diseases [17]. Previous studies have shown that FTZ could inhibit the development of atherosclerosis by reducing the degree of vascular restenosis in the atherosclerosis model by regulating the adiponectin signaling pathway and inhibiting the expression of inflammatory factors [17–19]. However, the effect and molecular mechanisms underlying FTZ in cardiac diseases, especially in DCM have not yet been widely reported.

Therefore, this study aimed to investigate the cardioprotective effect of FTZ and understand the mechanism underlying the beneficial effects of this traditional Chinses medicine (TCM) *in vivo* and *in vitro*.

## 2. Methods

### 2.1. Animals and Treatment

Male C57BL/6 mice (8–12 weeks old) were purchased from the Guangdong Medical Laboratory Animal Center (Guangzhou, China). Type 1 diabetes mellitus (T1DM) was induced in mice by intraperitoneal injection of streptozotocin (STZ) (Sigma-Aldrich, St. Louis, MO, USA) at a dose of 50 mg/kg/day body weight for 5 consecutive days [20], while control mice were injected with vehicle (0.1 M sodium citrate buffer, pH 4.5). Mice were defined as diabetic when glucose levels were ≥15 mM on an UltraEasy glucose meter (Johnson and Johnson, New Brunswick, NJ, USA) after two consecutive determinations under nonfasting conditions [21]. The diabetic mice were randomly allocated into three groups: control group (Con), diabetes group (DCM), and diabetes + FTZ group (DCM + FTZ). FTZ was diluted in 0.5% carboxymethyl cellulose-Na (CMC-Na) (Tianjin Zhiyuan Chemical Reagent Co., Ltd., Tianjin, China), and 3 g (crude drug)/kg/day FTZ was used for intragastric administration to mice every day [13, 14]. The Con group and DCM group were treated with the considerable volume of 0.5% CMC-Na via intragastric administration once a day. The preparation and quality analysis of FTZ extract were consistent with the protocol described previously [15, 22]. Mice were assessed and killed after 1 month after FTZ treatment [23]. All mice were provided with free access to food and water. The study was approved by the Guangdong Pharmaceutical University Ethics Committee on Animal Care (Approval N : GDPULACSPF2017314), and all procedures were carried out following the Guide for the Care and Use of Laboratory Animals (2011). Blood and heart tissue collection methods have been described previously [24].

### 2.2. Echocardiography

Mice were anesthetized with 1.5% isoflurane/oxygen, and transthoracic echocardiography was performed using the Vevo 2100 system (FUJIFILM VisualSonics, Toronto, Canada) to measure cardiac diameter and function as previously described [24]. The heart was imaged in the 2-dimensional parasternal short-axis view, and an M-mode echocardiogram of the midventricle was recorded at the level of the papillary muscles. Systolic parameters, including end-diastolic and end-systolic interventricular septal thicknesses (IVSd and IVSs), end-diastolic and -systolic left ventricular (LV) internal diameters (LVIDd and LVIDs), end-diastolic and -systolic left ventricular posterior wall thicknesses (LVPWd and LVPWs), LV fractional shortening (LVFS), and LV ejection fraction (LVEF) were measured from the M-mode image. Pulse-wave Doppler was used to assess the mitral valve flow (E/A ratio), as a reliable measure of diastolic function.

### 2.3. Immunoassay for Serum Parameters

Serum insulin, interleukin-1*β* (IL-1*β*), IL-6, IL-10, IL-12, tumor necrosis factor *α* (TNF-*α*), and C-C motif chemokine ligand 2 (CCL2) were tested by enzyme-linked immunosorbent assay (ELISA) using mouse immunoassay kits from Nanjing Jiancheng Bioengineering Institute (Nanjing, China) according to the manufacturer's protocol.

### 2.4. Cell Culture and Treatment

The murine fibroblast cell line C3H/10T1/2 (Clone 8) was purchased from the Cell Bank of Type Culture Collection, Chinese Academy of Sciences (Shanghai, China) and routinely cultured in Dulbecco's modification of Eagle's medium (DMEM) at 37°C in a humidified atmosphere containing 5% CO_2_ and 95% humidified atmosphere. C3H/10T1/2 cells were cultured onto 6-well plates overnight and then treated with transforming growth factor-*β*1 (TGF-*β*1) (PeproTech, Rocky Hill, NJ, USA; 50 ng/ml) [25] and FTZ extracts at different concentrations (1 *μ*g/ml, 10 *μ*g/ml, and 100 *μ*g/ml) for 12 h or 24 h. Total RNA and proteins of cells were collected from the cells for further testing.

### 2.5. Real-Time PCR

Total RNA was extracted from the ventricle tissues or cells using RNAiso Plus (TaKaRa, Tokyo, Japan) and reverse-transcribed into cDNA using the ReverTra Ace qPCR RT Master Mix with gDNA Remover (TOYOBO, Osaka, Japan). PCR primers were designed and synthesized by Sangon Biotech Co., Ltd. (Shanghai, China) (Table 1). Real-time PCR analysis was performed using the KOD SYBR qPCR Mix (TOYOBO) with LightCycle 480 (Roche, Basel, Switzerland), according to the manufacturer's instructions. The expression of target genes was determined by normalizing to the *Gapdh*.

### 2.6. Western Blot Analysis

Proteins were isolated from the ventricular homogenate or cells with lysis buffer (Beyotime Institute of Biotechnology, Shanghai, China) containing phenylmethylsulfonyl fluoride (PMSF) (Sigma-Aldrich) and subjected to Western blot, as described previously [24]. The antibodies used in this study were glyceraldehyde-3-phosphate dehydrogenase (GAPDH) and collagen type III (Col3al) (Proteintech, Rosemont, IL, USA), matrix metalloproteinase 2 (MMP2) and MMP9 (Abcam, Cambridge, MA, USA), *α*-SMA (Bioss, Beijing, China), cellular communication network factor 2 (CCN2) (Santa Cruz Biotechnology, Santa Cruz, CA, USA), and TNF-*α*, p-AKT (Ser473), AKT, p-ERK (Thr202/Tyr204), ERK, p-STAT3 (Tyr705), STAT3, and Caspase-3 (Cell Signaling Technology, Danvers, MA, USA).

### 2.7. Picrosirius Red, Hematoxylin-Eosin (H&E), and TUNEL Staining

Excised hearts were fixed in 4% paraformaldehyde, paraffin-embedded, and sectioned at 5 *μ*m thickness. The picrosirius red staining, H&E staining, and TUNEL were performed as described previously [24, 26].

### 2.8. Network Pharmacology Analysis

According to the parameters of absorption, distribution, metabolism, and excretion (ADME), the active ingredients of FTZ prescription were screened for in the traditional Chinese medicine system and analyzing platforms (TCMSP) (http://tcmspw.com/tcmsp.php) [27], the Bioinformatics Analysis Tool for Molecular mechANism of Traditional Chinese Medicine (BATMAN-TCM) (http://bionet.ncpsb.org/batman-tcm/) [28], and the database of natural product activity and species source (NPASS) (http://bidd2.nus.edu.sg/NPASS) [29]. The screening conditions were oral bioavailability (OB) greater than 30% and drug similarity (DL) greater than 0.18 [30]. Another gene list related to DCM was established after screening GeneCards (https://www.genecards.org). All genes and targets were submitted to the UniProt database to validate their gene names. The PPI networks, Gene Ontology (GO) functional annotation, and KEGG pathway analysis procedures have been described previously [31, 32].

### 2.9. Statistical Analysis

Continuous variables are expressed as mean ± standard deviation. ANOVA with Tukey's multiple comparisons test (equal variance) or the Kruskal-Wallis test, followed by Dunn's multiple comparison test (unequal variance) was used for multiple comparisons. The GraphPad Prism 7 (GraphPad Software Inc., La Jolla, CA, USA) was used for data analysis. Two-sided tests were used throughout the study, and *p* < 0.05 was considered statistically significant.

## 3. Results

### 3.1. FTZ Attenuated the Increase in Blood Glucose, Decrease in Serum Insulin, and Body Weight Loss in STZ-Induced Diabetic Mice

**Figure 1:**
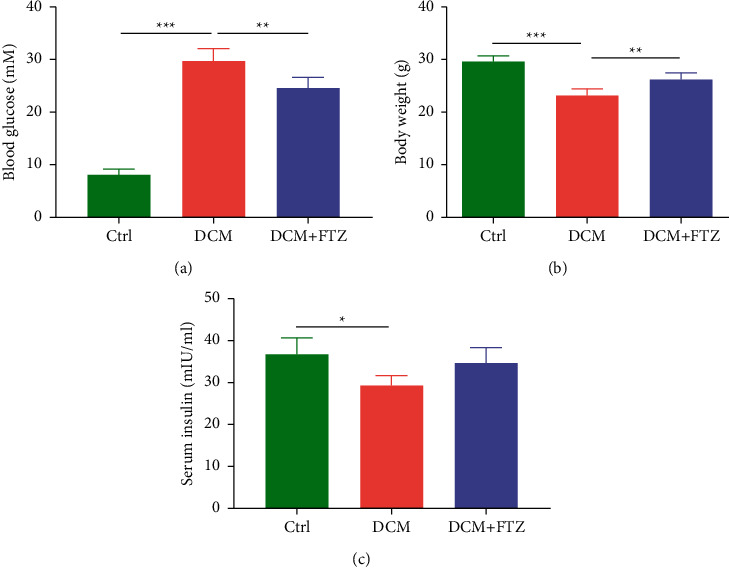
FTZ ameliorated the changes in blood glucose, serum insulin, and body weight in diabetic mice. Mice were treated with streptozotocin (STZ) (50 mg/kg/d, injected intraperitoneally every day for 5 consecutive days) followed by treatment with or without FTZ (3 g/kg/d, administrated intragastrically every day for one month). At the end of the experiment, blood glucose (a), body weight (b), and serum insulin (c) were tested. *n* = 5. ^*∗*^*p* < 0.05, ^*∗∗*^*p* < 0.011, and ^*∗∗∗*^*p* < 0.001.

A diabetes model was established by intraperitoneal injection of STZ in mice. To confirm the effectiveness of this diabetes model, we tested the blood glucose, body weight, and serum insulin levels at the end of the experiment. As shown in Figure 1, compared with the control group, the level of blood glucose significantly increased in the STZ-treated group (DCM group) (29.68 ± 2.43 mM versus 7.98 ± 1.12 mM, *p* < 0.001), whereas body weight and serum insulin level were significantly lower in the DCM group (body weight: 23.14 ± 1.24 g versus 29.5 ± 1.14 g, *p* < 0.001; serum insulin: 29.27 ± 2.37 mIU/ml versus 36.6 ± 3.94 mIU/ml, *p* < 0.05). After treatment with FTZ for 4 weeks, the level of blood glucose notably decreased (24.54 ± 2.04 mM versus 29.68 ± 2.43 mM, *p* < 0.01), but the body weight markedly increased (26.16 ± 1.29 g versus 23.14 ± 1.24 g, *p* < 0.01) (Figures 1(a) and 1(b)). Although there was no significant difference in the serum insulin level between the DCM and the DCM + FTZ groups, an increasing trend of serum insulin was observed in the DCM + FTZ group (Figure 1(c)). FTZ-only treatment did not affect body weight or blood glucose level (Supplemental Figure 1(a)).

### 3.2. FTZ Improved the Cardiac Function in Diabetic Mice

**Figure 2:**
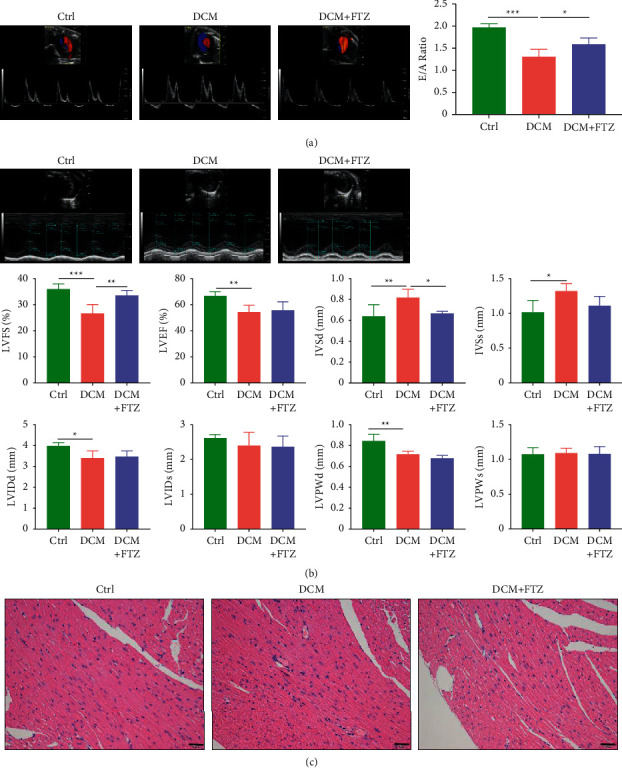
FTZ improved the cardiac function in diabetic mice. (a) The early to late diastolic peak velocity (E/A) ratio was detected by Doppler echocardiography. (b) The ventricular wall thickness was detected by M-mode echocardiography. (c) H&E staining. Bar = 50 *μ*m. n = 5. ^*∗*^*p* < 0.05, ^*∗∗*^*p* < 0.01, and ^*∗∗∗*^*p* < 0.001.

DCM initially manifests as an isolated diastolic dysfunction, but with time progressing to systolic dysfunction [1]. Therefore, heart function was measured using echocardiography. The E/A ratio, a marker of cardiac diastolic function, was decreased in the DCM group compared to the control group (1.31 ± 0.16 versus 2.02 ± 0.08, *p* < 0.001) (Figure 2(a)). In addition, LVFS and LVEF, markers of cardiac systolic performance, significantly decreased in the DCM group compared to the control group (LVFS: 26.68 ± 3.42% versus 36 ± 1.98%, *p* < 0.001; LVEF: 54.48 ± 5.13% versus 66.91 ± 3.03%, *p* < 0.01) (Figure 2(b)). Increased IVSd and IVSs as well as decreased LVIDd and LVPWd were also observed in the DCM group (IVSd: 0.82 ± 0.08 mm versus 0.64 ± 0.11 mm, *p* < 0.01; IVSs: 1.32 ± 0.11 mm versus 1.02 ± 0.17 mm, *p* < 0.05; LVIDd: 3.39 ± 0.35 mm versus 3.97 ± 0.17 mm, *p* < 0.05; LVPWd: 0.72 ± 0.03 mm versus 0.84 ± 0.07 mm, *p* < 0.01) (Figure 2(b)). Notably, FTZ treatment increased the E/A ratio (1.59 ± 0.14 versus 1.31 ± 0.16, *p* < 0.05), LVFS (33.62 ± 1.90% versus 26.68 ± 3.42%, *p* < 0.01), and reduced IVSd (0.67 ± 0.02 mm versus 0.82 ± 0.08 mm, *p* < 0.05) in diabetic mice. H&E staining revealed that there were a large number of inflammatory cells infiltrated and dense myocardial fibers in heart of the DCM group, and FTZ treatment improved the conditions in diabetic mice (Figure 2(c)). Treatment with FTZ alone did not affect cardiac structure or function (Supplemental Figures 1(b) and 1(c)). These results indicate that FTZ can significantly improve left ventricular structure and function in diabetic animals.

### 3.3. FTZ Treatment Inhibited the Inflammation in Diabetic Mice

**Figure 3:**
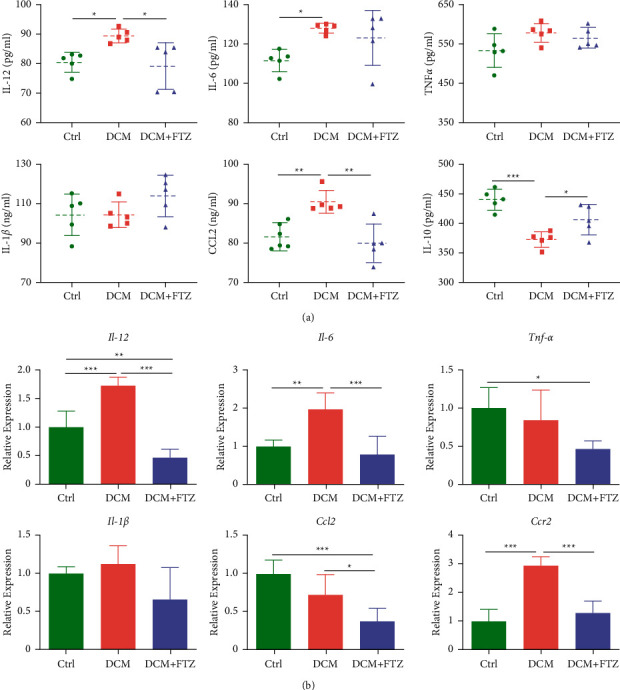
FTZ inhibited the inflammation in diabetic mice. (a) Serum levels of IL-12, IL-6, TNF-*α*, IL-1*β*, CCL2, and IL-10 were measured by ELISA. (b) The myocardial mRNA levels of *IL-12*, *IL-6*, *Tnf-α*, *IL-1β*, *Ccl2*, and *Ccr2* were tested by Q-PCR. *n* = 5. ^*∗*^*p* < 0.05, ^*∗∗*^*p* < 0.01, and ^*∗∗∗*^*p* < 0.001.

Inflammation plays an important role in the development of DCM [1,7]. First, we measured the levels of inflammatory factors in the peripheral blood. As shown in Figure 3(a), the serum levels of IL-6, IL-12, and CCL2 increased in the DCM group compared to those in the control group (IL-6:127.87 ± 2.49 pg/ml versus 111.41 ± 5.58 pg/ml, *p* < 0.05; IL-12 : 89.37 ± 2.3 pg/ml versus 80.45 ± 3.4 pg/ml; *p* < 0.05; CCL2: 90.45 ± 2.88 ng/ml versus 81.58 ± 3.59 ng/ml; *p* < 0.01). Notably, FTZ significantly reduced the increased levels of IL-12 and CCL2 in diabetic mice (IL-12 : 79.13 ± 7.93 pg/ml versus 89.37 ± 2.3 pg/ml; *p* < 0.05; CCL2: 79.91 ± 4.89 ng/ml versus 90.45 ± 2.88 ng/ml; *p* < 0.01). Furthermore, the serum level of IL-10, an important anti-inflammatory factor, significantly decreased in the DCM group compared to the control group (373.04 ± 13.23 pg/ml versus 439.88 ± 17.39 pg/ml; *p* < 0.001). Notably, FTZ treatment markedly increased IL-10 levels in diabetic mice (406.42 ± 25.81 pg/ml versus 373.04 ± 13.23 pg/ml; *p* < 0.05) (Figure 3(a)). There were no significant differences in the levels of IL-1*β* and TNF-*α* among the three groups.

Next, we tested the levels of inflammation-related genes in heart tissues. The results of real-time PCR showed that *IL-12* (*p* < 0.001), *IL-6* (*p* < 0.01), and *Ccr2* (*p* < 0.001) were considerably higher in the DCM group than in the control group and FTZ significantly decreased the levels of *IL-12* (*p* < 0.001), *IL-6* (*p* < 0.001), *Ccl2* (*p* < 0.05), and *Ccr2* (*p* < 0.001) in the diabetic heart (Figure 3(b)). Moreover, the levels of *IL-12* (*p* < 0.01), *Tnf-α* (*p* < 0.05), and *Ccl2* (*p* < 0.001) significantly decreased in the DCM + FTZ group compared to those in the control group (Figure 3(b)). FTZ-only treatment did not affect the levels of inflammation-related genes *IL-6* and *Ccl2* in the heart tissue (Supplemental Figure 1(d)). These results demonstrated that FTZ treatment can ameliorate the systemic and cardiac inflammation in diabetic cardiomyopathy.

### 3.4. FTZ Alleviated the Cardiac Fibrosis in Diabetic Mice

**Figure 4:**
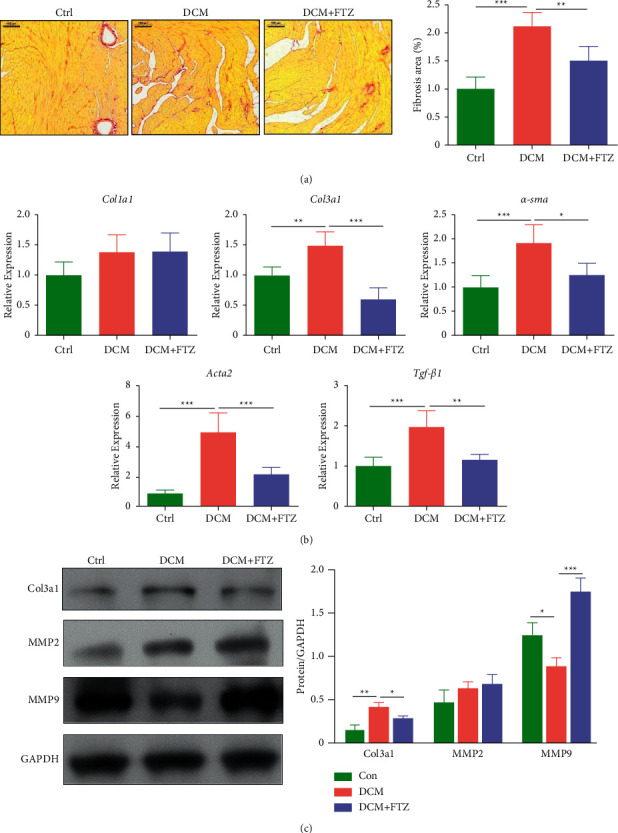
FTZ alleviated diabetes-induced cardiac fibrosis. (a) Histopathological features of collagen deposition by Sirius Red staining of heart sections from mice model and quantification of Sirius Red staining. Bar = 100 *μ*m. (b) The myocardial mRNA expressions of *Col1al*, *Col3al*, *α-sma*, *Acta2*, and *Tgf-β1* were tested by Q-PCR. *n* = 5. (c) The protein expressions of Col3a1, MMP2, and MMP9 in hearts were tested by Western blot. *n* = 3. ^*∗*^*p* < 0.05, ^*∗∗*^*p* < 0.01, and ^*∗∗∗*^*p* < 0.001.

Cardiac fibrosis is a major pathological characteristic of DCM [1, 33]. Picrosirius red staining showed that collagen deposition significantly increased in the DCM group compared to that in the control group (2.12 ± 0.25% versus 1.01 ± 0.2%, *p* < 0.001), while FTZ treatment significantly decreased collagen deposition in the hearts of diabetic mice (1.51 ± 0.24% versus 2.12 ± 0.25%, *p* < 0.01) (Figure 4(a)). Next, we tested the changes in fibrotic genes in heart tissues. As shown in Figure 4(b), the expression levels of *Col3al* (*p* < 0.01), *Ccn2* (*p* < 0.001), *Acta2* (encoding the protein *α*-SMA) (*p* < 0.001), and *Tgf-β1* (*p* < 0.001) genes increased in the DCM group compared to those in the control group. In contrast, FTZ treatment significantly reversed the increased levels of *Col3al* (*p* < 0.05), *Ccn2* (*p* < 0.001), *Tgf-β1* (*p* < 0.01), and *Acta2* (*p* < 0.05) genes in diabetic mice (Figure 4(b)). Consistent with this, the DCM group showed increased levels of Col3al protein compared to the control group, and FTZ treatment restored the expression of Col3al to a level comparable to that of the control (Figure 4(c)). In addition, treatment with only FTZ did not affect the expression of *Col3al* or *Tgf-β1* in the cardiac tissue (Supplemental Figure 1(e)).

Due to the vital role of MMP2 and MMP9 in the degradation of Col3al [34], we assessed the changes in MMP2 and MMP9 in the hearts of different groups. As shown in Figure 4(c), the level of MMP2 showed an increasing trend in the DCM group compared to the control group, and no change was observed between the DCM group and the DCM + FTZ group, while the protein level of MMP9 significantly decreased in the hearts of diabetic mice but was reversed by FTZ treatment (Figure 4(c)).

### 3.5. FTZ Treatment Decreased the Apoptosis in the Heart of Diabetic Mice

**Figure 5:**
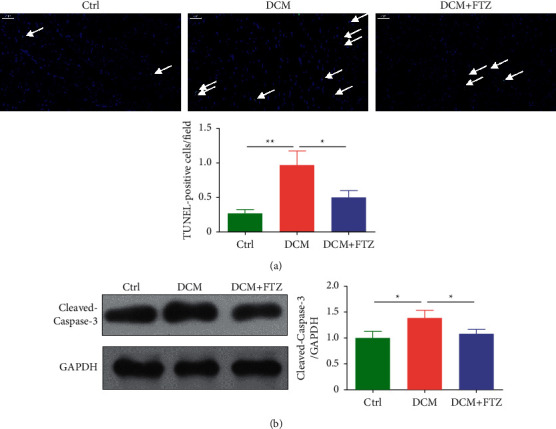
FTZ decreased diabetes-induced cardiac apoptosis. (a) TUNEL staining and quantification. Bar = 50 *μ*m. *n* = 5. (b) The protein expressions of cleaved-Caspase-3 in hearts were tested by Western blot. *n* = 3. ^*∗*^*p* < 0.05 and ^*∗∗*^*p* < 0.01.

We tested the apoptosis in the heart using TUNEL staining and Western bolt. The results showed that the apoptosis of heart was enhanced in the DCM group while FTZ inhibited the increased apoptosis in diabetic mice, as seen in the TUNEL-positive cells and the changes in cleaved-Caspase-3 (Figure 5). These data indicate that FTZ can ameliorate the apoptosis in heart of diabetic mice.

### 3.6. Network Pharmacology Analysis of the Potential Targets of FTZ in the Treatment of Diabetic Cardiomyopathy

**Figure 6:**
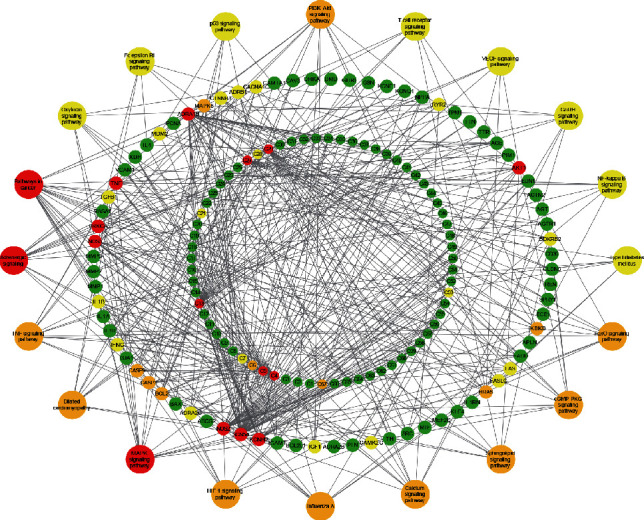
Component-target-pathway network of FTZ formula in the treatment of DCM. Nodes in red are considered as important, and the green nodes are considered as less important.

A total of 67 components and 76 genes were identified as the main components and target genes of FTZ in the treatment of DCM, respectively. The combination of component-target (C-T) and target-pathway (T-P) databases constituted a component-target-pathway (C-T-P) network (Figure 6), providing us with an overview of the therapeutic effects of FTZ.

The inner cycle represents the main component of the FTZ. Nodes painted in red are key components which interact with a larger number of targets. Among the 67 components, neocryptotanshinone ii from *Salviae Miltiorrhizae Radix et Rhizoma*, quercetin from *Ligustri Lucidi Fructus*, *Eucommiae Cortex*, *Notoginseng Radix et Rhizome,* and *Coptidis Rhizoma*, epiquinidine from *Eucommiae Cortex*, miltirone from *Salviae Miltiorrhizae Radix et Rhizoma*, and luteolin from *Ligustri Lucidi Fructus* and *Salviae Miltiorrhizae Radix et Rhizoma* were regarded as the key components of FTZ in the treatment of DCM (Figure 6; Supplemental Table 1).

The middle cycle represents the main target proteins regulated by FTZ. When painted in red, the corresponding targets are regulated by more components and participate in more pathways. The analysis identified AKT1, TNF, SCN5A, ADRA1B, NOS2, KCNH2, NOS3, and PRKCA as key proteins regulated by the FTZ formula (Figure 6).

The outer cycle represents the top 20 enriched pathways. Pathways containing the most target genes were painted in red; of note, the MAPK, PI3K-AKT, and TNF pathways are important pathways (Figure 6).

### 3.7. FTZ Inhibited the Activity of AKT, ERK, and STAT3 in Diabetic Mice

**Figure 7:**
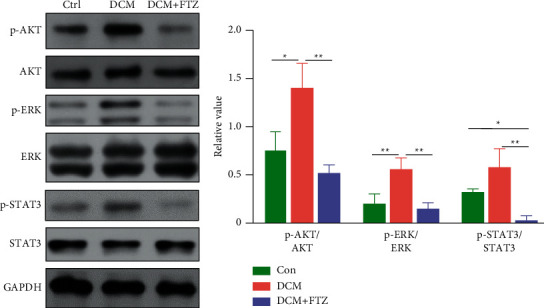
Effects of FTZ on the activity of AKT, ERK, and STAT3 in hearts of DCM. The proteins of p-AKT, AKT, p-ERK, ERK, p-STAT3, and STAT3 in hearts were tested by Western blot. *n* = 3. ^*∗*^*p* < 0.05 and ^*∗∗*^*p* < 0.01.

Based on the results of the above network pharmacology and other reports [35–37], we tested the activity changes of AKT, ERK, and STAT3 in the hearts of mice from different groups. As shown in Figure 6, the phosphorylated forms of AKT, ERK, and STAT3 were significantly augmented in the hearts of the DCM group compared to those in the control group. After treatment with FTZ, the activity of AKT, ERK, and STAT3 was markedly reduced in the hearts of diabetic mice (Figure 7). Moreover, the total protein levels of AKT, ERK, and STAT3 were not significantly different among different groups.

### 3.8. FTZ Inhibited the Expressions of Fibrotic Factors in C3H/10T1/2 Cells

**Figure 8:**
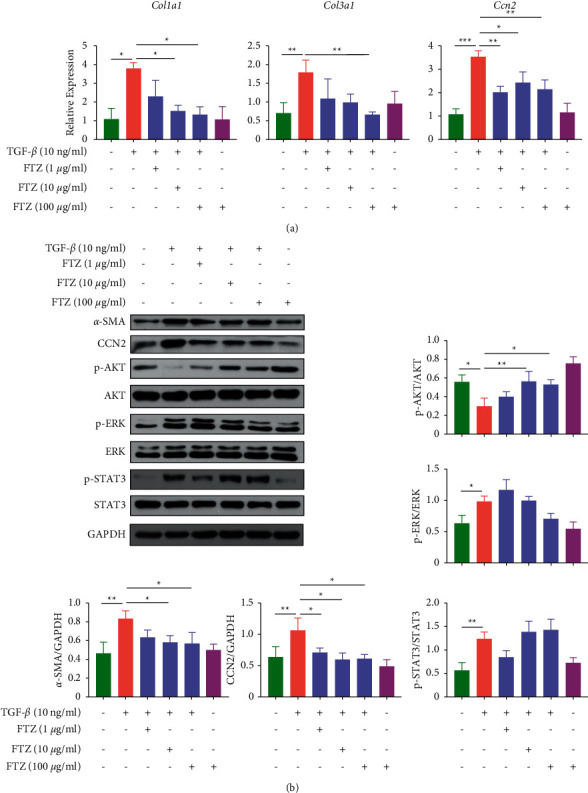
FTZ inhibited the expressions of fibrotic factors in C3H/10T1/2 cells. C3H/10T1/2 cells were treated with TGF-*β*1 (50 ng/ml) and different FTZ (1 *μ*g/ml, 10 *μ*g/ml, and 100 *μ*g/ml) for 12 h or 24 h. (a) The mRNA levels of *Col1al*, *Col3al*, and *Acta2* in cells were tested by Q-PCR. (b) The protein changes of *α*-SMA, CTGF, p-AKT, AKT, p-ERK, ERK, p-STAT3, and STAT3 in cells were tested by Western blot. *n* = 3. ^*∗*^*p* < 0.05, ^*∗∗*^*p* < 0.01, and ^*∗∗∗*^*p* < 0.001.

We subsequently tested these findings *in vitro*. We found that the mRNA level of TGF-*β*1 significantly increased in the hearts of the DCM group (Figure 4(b)), indicating that TGF-*β*1 plays an important role in DCM. Therefore, 50 ng/ml TGF-*β*1 was used to induce fibrosis in the *in vitro* cell model [25]. As shown in Figures 7(a) and 7(b), TGF-*β*1 treatment significantly increased the mRNA levels of *Col1al* (*p* < 0.05), *Col3al* (*p* < 0.01), and *Ccn2* (*p* < 0.001) and the protein expression of *α*-SMA and CCN2. After treatment with FTZ, the levels of fibrotic factors significantly decreased in a concentration-dependent manner compared with that in TGF-*β*1-treated cells (Figures 8(a) and 8(b)).

Then we detected changes in the activity of AKT, ERK, and STAT3 in the cells. As shown in Figure 8(b), the ratio of p-AKT/AKT significantly decreased, while the ratios of p-ERK/ERK and p-STAT3/STAT3 notably increased in the TGF-*β*1-treated group compared to the control group. After treatment with FTZ, the decreased activity of AKT was significantly reversed, and a decreasing trend in ERK was observed (Figure 8(b)).

## 4. Discussion

Cardiomyopathy, an independent complication of diabetes, occurs in the absence of other heart diseases [1]. It causes at least a four- to fivefold increase in the risk of developing heart failure in diabetes patients [1, 38]. Although the pathophysiology of DCM is still under investigation, the underlying molecular mechanisms are not fully understood [39], and there is thus currently no specific drug for its treatments. In this study, we evaluated the cardioprotective effect of FTZ in an STZ-induced DCM mouse model. Our findings showed that FTZ treatment decreased the interventricular septal thickness and improved systolic and diastolic function in the DCM model. Moreover, FTZ attenuated the accumulation of interstitial collagens and downregulated the expression of fibrosis-related genes in the hearts of DCM mice. Treatment with FTZ inhibited systemic inflammation and cardiac inflammation caused by diabetes. Furthermore, FTZ significantly inhibited the activities of AKT1, ERK, and STAT3. Our data indicate that FTZ may serve as a therapeutic agent for treating DCM.

The principal approach for treating DM is to control the blood glucose levels. In this study, STZ-induced diabetic mice exhibited a notable increase in blood glucose levels concomitantly with decreased body weight, as previously reported [5]. Consistent with a previous study showing that FTZ reduced blood glucose levels in a model of metabolic syndrome [14], our results demonstrated that treatment with FTZ significantly improved blood glucose levels in T1DM mice. This may be due to the protective effect of FTZ on pancreatic *β*-cells [13]. These data indicate that FTZ can regulate blood glucose levels via various mechanisms.

Various studies have shown that inflammation is a key pathogenic feature and plays a crucial role in DCM [1, 5, 7, 8]. In the setting of diabetes, hyperglycemia, elevated angiotensin II levels, and other upregulated proinflammatory factors promote the accumulation and infiltration of proinflammatory cells, such as macrophages and lymphocytes into the lesion site. These inflammatory cells and other noninflammatory cells secrete cytokines such as IL-1*β*, IL-6, TNF-*α*, and TGF-*β*, which can induce or exacerbate the cardiac injury, leading to further adverse remodeling [1]. Studies have shown that proinflammatory cytokines, such as IL-1*β*, IL-6, and TNF-*α*, are involved in the development of contractile dysfunction that impairs cardiac contractility in mice [40–42]. Moreover, the deletion of IL-6 could alleviate interstitial fibrosis in STZ-induced DCM [43]. In addition, the treatment of diabetic rats with an anti-TNF-*α* monoclonal antibody suppressed myocardial inflammation and fibrosis [44]. The diabetic model mice in the present study exhibited increased serum levels of IL-12 and IL-6 and increased mRNA levels of *IL-12* and *IL-6* in the heart. In line with previous studies showing that the cardiac level of IL-10, an important anti-inflammatory cytokine, significantly decreased in diabetic animals [45, 46], we found that the serum level of IL-10 significantly increased in diabetic mice. These data indicate that the imbalance between the proinflammatory cytokines and anti-inflammatory cytokines plays a vital role in DCM, and the rebalance of the inflammatory level might have direct cardioprotective effects on diabetes. In this study, FTZ treatment markedly decreased the levels of proinflammatory cytokines (IL-12, IL-6, CCL2, and TNF-*α*) and significantly increased the levels of the anti-inflammatory cytokine IL-10, indicating that TCM has unique advantages in regulating the rebalance of inflammation. And the results of this study also indicate that inflammation is the biological feature of “Zhuo” in TCM, and the “Hua Zhuo” effect of FTZ is to inhibit the inflammatory response.

Recent studies have shown that STAT3 pathway activation participates in the pathophysiological development of DCM, and inhibition of STAT3 attenuates cardiomyopathy in STZ-induced T1DM [37, 47]. In our study, we found that the phosphorylated form of STAT3 was promoted in the DCM model and FTZ treatment retarded this activity, indicating that FTZ ameliorated DCM, at least partially by inhibiting the STAT3 pathway. In addition, the AKT signaling pathway is involved in the growth, metabolism, and apoptosis of myocardial cells. Previous studies have shown that the activity of AKT is augmented in DCM, and inhibition of the AKT pathway can alleviate cardiomyocyte apoptosis and improve cardiac function [36, 48]. Our network pharmacology results showed that the AKT signaling pathway was a potential target signaling pathway of FTZ in DCM treatment. Moreover, at the protein level, we found that the activity of AKT was enhanced in DCM and was significantly alleviated in FTZ treatment. We also found that ERK was activated in the hearts of the DCM group and was markedly inhibited in the DCM + FTZ group. These results are in line with previous research showing that the AKT and ERK pathways are involved in the pathogenesis of DCM [35]. AKT regulates ERK activity in cardiomyocytes [35]. However, the changes in these proteins in C3H/10T1/2 cells were not in accordance with those in heart tissues. The possible reasons are as follows: (1) The mechanism of cardiac fibrosis in T1DM is sophisticated, including not only the increased profibrotic factors but also hyperglycemia, increased advanced glycosylation end products (AGEs), and an overactive inflammatory response [1, 6]. In the cell experiment, we treated cells with only the profibrotic factor TGF-*β*1 to test the antifibrotic effect of FTZ *in vitro*. (2) The heart contains various cell types, including cardiomyocytes, fibroblasts, endothelial cells, macrophages, and neutrophils [49,50]. Fibroblasts account for approximately 10% of the total cells in the adult mouse heart [51]. Under diabetic conditions, the changes in STAT3, AKT, and ERK may be more different in nonfibroblasts than in fibroblasts. (3) In the mouse model, FTZ may be metabolized after entering the blood, and the activity of some components may increase, while that of others may decrease. We directly treated cells with FTZ *in vitro*. Further studies will test the effects of other stimuli, such as high glucose and AGEs, on the changes in these proteins in cardiomyocytes and fibroblasts and the effect of medicated serum of FTZ on fibroblasts.

The CCL2-CCR2 axis is critical in the recruitment of inflammatory monocytes/macrophages into damaged tissues [52]. High glucose levels can induce the production of CCL2 in heart cells both *in vivo* and *in vitro* [53, 54]. In addition, the expression of CCR2 was upregulated in the hearts of the STZ-induced DCM model [55]. CCR2 knockout significantly improved diabetic cardiac dysfunction and fibrosis by inhibiting CCR2-induced inflammation and oxidative stress [55]. In our study, we found that the serum levels of CCL2 and the cardiac mRNA level of *Ccr2* significantly increased in diabetic mice, indicating that macrophages were accumulated in the heart and became polarized to the M1 state in diabetic mice [23, 55]. Treatment with FTZ in the present study markedly decreased the levels of CCL2 and CCR2. These data indicate that FTZ may reduce the secretion of CCL2 to inhibit the cardiac infiltration of proinflammatory macrophages, resulting in a decrease in CCR2.

Cardiac fibrosis, as one of the characteristics of an advanced stage of DCM, can lead to increased myocardial stiffness, reduced compliance, and ultimate cardiac dysfunction [6]. Under physiological circumstances, cardiac fibroblasts synthesize small amounts of collagen to maintain ECM homeostasis. However, under diabetic conditions, AGEs generated by the exposure of proteins and lipids to high glucose levels crosslink ECM proteins to impair ECM degradation [1]. Besides, altered cardiac mechanics result in the release of stimuli, including TGF-*β*, TNF, angiotensin II, and various interleukins, which transform fibroblasts into myofibroblasts (active form) [56]. In our study, we found that ECM accumulated in the myocardial interstitium of the DCM model and that the mRNA level of *Tgf-β1* increased in the heart tissue of diabetic mice. Treatment with FTZ reduced the level of *Tgf-β1* and alleviated cardiac fibrosis, which may contribute to the improvement of cardiac function by FTZ treatment.

MMPs are the fundamental proteases responsible for the degradation of ECM components and play an integral role in maintaining the balance between anabolism and catabolism of the ECM [57]. As the largest and most complex member of the MMP family, MMP9 is essential for ECM degradation [58]. However, research regarding the expression of MMP9 in the hearts of the T1DM model is contradictory. Some studies have reported that the expression of MMP9 is consistent with cardiac fibrosis and markedly increased in the myocardium of T1DM [59–61]. However, other studies have shown that the level of MMP9 is negatively correlated with fibrosis and downregulated in the hearts of diabetic animals [62, 63]. In our study, we found that the protein level of MMP9 significantly reduced in the cardiac tissue of the DCM group, while it was markedly upregulated in the hearts of the DCM + FTZ group. In addition, Bollano et al. reported that the protein expression of MMP2 was significantly decreased in the heart tissue of diabetes rat, while MMP9 was unchanged [64]. However, another study has shown that MMP2 was elevated while MMP9 was decreased in the heart of diabetes rat [65]. These data indicate that the changing trends of MMP2 and MMP9 are not always the same or similar in diabetes hearts. In our study, the protein level of MMP2 did not significantly change among the three groups. The data indicate that downregulated MMP9 not MMP2 may partly result in the increased protein level of Col3al, contributing to cardiac fibrosis in DCM, and FTZ alleviated myocardial fibrosis at least partially by increasing the expression of MMP9.

The network pharmacology strategy provides new methods for revealing the complex and understanding the effects of TCM prescriptions [66]. The cardinal principles of TCM to diagnose and treat diseases are a holistic view and syndrome differentiation [67]. Network pharmacology, characterized by holistic, systemic, and drug-oriented interactions, is in accordance with the basic characteristics of TCM, which is a new discipline, which uncovers the possible regulatory effects of compound drugs on the biological network at the systemic level, and establishes a bridge between TCM and modern medicine [66, 67]. In this study, we analyzed the components of FTZ, the potential molecules, and corresponding signaling pathways in DCM treated with FTZ using network pharmacology. The results of network pharmacology provided the basis for the following mechanistic study.

In summary, we assessed the cardioprotective effects of FTZ on the treatment of DCM. FTZ reduced hyperglycemia, improved cardiac function, and attenuated inflammation and fibrosis. Furthermore, FTZ treatment modulated the AKT-ERK and STAT3 pathways. Thus, FTZ has therapeutic potential in the treatment and/or prevention of DCM. However, further studies are needed to determine the exact molecular mechanisms underlying the therapeutic potential of FTZ.

## Figures and Tables

**Table 1:** Primer sequences used for real-time PCR.

Gene (mouse)	Forward sequence	Reverse sequence
*IL-1β*	CAACTGTTCCTGAACTCAACT	ATCTTTTGGGGTCCGTCAACT
*IL-6*	AGTTGCCTTCTTGGGACTGA	TCCACGATTTCCCAGAGAAC
*IL-12*	AAATGAAGCTCTGCATCCTGC	TCACCCTGTTGATGGTCACG
*Tnf-α*	CTCACACTCAGATCATCTTCT	GCTACGACGTGGGCTACAG
*Ccl2*	AATTAAAAACCTGGATCGG	TTAGCTTCAGATTTACGGGT
*Ccr2*	TCCACGGCATACTATCAACATC	AAGGCTCACCATCATCGTAG
*Col1a1*	AACTCCCTCCACCCCAATCT	CCATGGAGATGCCAGATGGTT
*Col3a1*	ACGTAAGCACTGGTGGACAG	GGAGGGCCATAGCTGAACTG
*Acta2*	AAGTCCCAGACATCAGGGAGT	ATCGGATACTTCAGCGTCAGG
*Tgf-β1*	CTCCCGTGGCTTCTAGTGC	CCTTAGTTTGGACAGGATCTG
*Ccn2*	AGTGTCTTCGGTGGGTCGGTGT	GGCAGTTGGCTCGCATCATAG
*Gapdh*	GGTCATCCATGACAACTT	GGGGCCATCCACAGTCTT

## Data Availability

The data used to support the findings of this study are included within the article.

## Supplementary Materials

Supplementary Materials *Supplemental Figure 1*. The effect of FTZ on mice. After one month of treatment with FTZ, the body weight and blood glucose of mice were tested (A) and the cardiac functions of mice were detected by echocardiography (B, C). The mRNA levels of inflammatory factors (*IL-*6 and Ccl2) (D) and fibrotic factors (Col3al and Tgf-*β*1) (E) were tested by Q-PCR. *n* = 3–5. *Supplemental Table 1*. The main components of the FTZ formula.
