# Supplementary material for: FTZ Ameliorates Diabetic Cardiomyopathy by Inhibiting Inflammation and Cardiac Fibrosis in the Streptozotocin-Induced Model
Source: Evid Based Complement Alternat Med. 2021 Sep 28;2021:5582567. doi: 10.1155/2021/5582567 (PMC8492284; doi:10.1155/2021/5582567)
Supplement: Supplementary Materials — Supplemental Figure 1. The effect of FTZ on mice. After one month of treatment with FTZ, the body weight and blood glucose of mice were tested (A) and the cardiac functions of mice were detected by echocardiography (B, C). The mRNA levels of inflammatory factors (IL-6 and Ccl2) (D) and fibrotic factors (Col3al and Tgf-β1) (E) were tested by Q-PCR. n = 3–5. Supplemental Table 1. The main components of the FTZ formula. [file 5582567.f1.docx]

**Supplemental materials**

**Supplemental Figure 1**

**
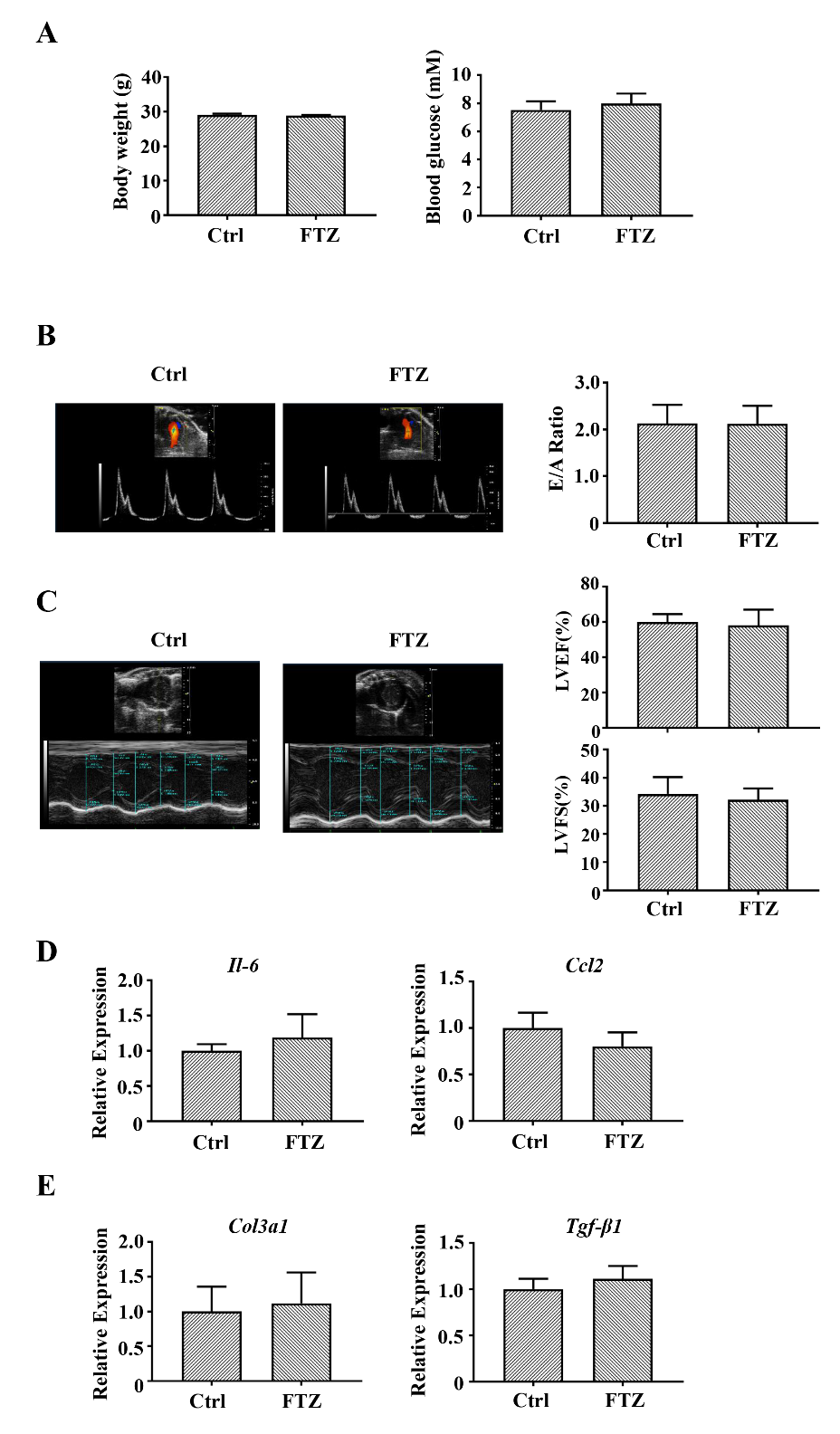
**

**Supplemental Figure 1**. The effect of FTZ on mice. After one month treatment with FTZ, the body weight and blood glucose of mice were tested (A) and the cardiac functions of mice were detected by Echocardiography (B, C). The myocardial mRNA levels of the inflmammory factors (*Il-6* and *Ccl2*) (D) and the fibrotic factors (*Col3al* and *Tgf-β1*) (E) were tested by Q-PCR. n=3-5.

**Supplemental Table 1. The main components of FTZ formula**

| No. | Name | OB/% | DL | Herb |
| --- | --- | --- | --- | --- |
| C1 | taxifolin | 57.84 | 0.27 | *Ligustri lucidi Fructus* |
| C2 | syringaresinol diglucoside_qt | 83.12 | 0.80 | *Ligustri lucidi Fructus* |
| C3 | Lucidumoside D_qt | 54.41 | 0.47 | *Ligustri lucidi Fructus* |
| C4 | quercetin | 46.43 | 0.28 | *Ligustri lucidi Fructus,Eucommiae Cortex,Notoginseng Radix et Rhizome,Coptidis Rhizoma* |
| C5 | luteolin | 36.16 | 0.25 | *Ligustri lucidi Fructus,Salviae miltiorrhizae radix et Rhizoma* |
| C6 | kaempferol | 41.88 | 0.24 | *Ligustri lucidi Fructus、Eucommiae Cortex* |
| C7 | beta-sitosterol | 36.91 | 0.75 | *Ligustri lucidi Fructus、Eucommiae Cortex、Notoginseng radix et Rhizoma* |
| C8 | 3β-acetoxyatractylone | 54.07 | 0.22 | *Atractylodis macrocephalae Rhizoma* |
| C9 | Ar-Curcumene | 52.34 | 0.65 | *Atractylodis macrocephalae Rhizoma* |
| C10 | Yangambin | 57.53 | 0.81 | *Eucommiae Cortex* |
| C11 | (+)-medioresinol | 87.19 | 0.62 | *Eucommiae Cortex* |
| C12 | medioresinol | 87.19 | 0.62 | *Eucommiae Cortex* |
| C13 | Epiquinidine | 68.22 | 0.40 | *Eucommiae Cortex* |
| C14 | Erythraline | 49.18 | 0.55 | *Eucommiae Cortex* |
| C15 | (+)-Eudesmin | 33.29 | 0.62 | *Eucommiae Cortex* |
| C16 | 8-Hydroxypinoresinol | 92.43 | 0.55 | *Eucommiae Cortex* |
| C17 | Tabernemontanine | 58.67 | 0.61 | *Eucommiae Cortex* |
| C18 | Dehydrodiconiferyl alcohol 4,gamma'-di-O-beta-D-glucopyanoside_qt | 51.44 | 0.40 | *Eucommiae Cortex* |
| C19 | Helenalin | 77.01 | 0.19 | *Eucommiae Cortex* |
| C20 | hirsutin_qt | 49.81 | 0.37 | *Eucommiae Cortex* |
| C21 | beta-carotene | 37.18 | 0.58 | *Eucommiae Cortex* |
| C22 | Syringetin | 36.82 | 0.37 | *Eucommiae Cortex* |
| C23 | Pinoresinol Dimethyl Ether | 33.29 | 0.62 | *Eucommiae Cortex* |
| C24 | sugiol | 36.11 | 0.28 | *Salviae miltiorrhizae radix et Rhizoma* |
| C25 | cryptotanshinone | 52.34 | 0.40 | *Salviae miltiorrhizae radix et Rhizoma* |
| C26 | Salvilenone | 30.38 | 0.38 | *Salviae miltiorrhizae radix et Rhizoma* |
| C27 | Miltirone | 38.76 | 0.25 | *Salviae miltiorrhizae radix et Rhizoma* |
| C28 | tanshinone iia | 49.89 | 0.40 | *Salviae miltiorrhizae radix et Rhizoma* |
| C29 | neocryptotanshinone ii | 39.46 | 0.23 | *Salviae miltiorrhizae radix et Rhizoma* |
| C30 | deoxyneocryptotanshinone | 49.40 | 0.29 | *Salviae miltiorrhizae radix et Rhizoma* |
| C31 | Dehydrotanshinone II A | 43.76 | 0.40 | *Salviae miltiorrhizae radix et Rhizoma* |
| C32 | 3α-hydroxytanshinoneⅡa | 44.93 | 0.44 | *Salviae miltiorrhizae radix et Rhizoma* |
| C33 | 4-methylenemiltirone | 34.35 | 0.23 | *Salviae miltiorrhizae radix et Rhizoma* |
| C34 | dan-shexinkum d | 38.88 | 0.55 | *Salviae miltiorrhizae radix et Rhizoma* |
| C35 | danshenspiroketallactone | 50.43 | 0.31 | *Salviae miltiorrhizae radix et Rhizoma* |
| C36 | dihydrotanshinlactone | 38.68 | 0.32 | *Salviae miltiorrhizae radix et Rhizoma* |
| C37 | dihydrotanshinoneⅠ | 45.04 | 0.36 | *Salviae miltiorrhizae radix et Rhizoma* |
| C38 | epidanshenspiroketallactone | 68.27 | 0.31 | *Salviae miltiorrhizae radix et Rhizoma* |
| C39 | C09092 | 36.07 | 0.25 | *Salviae miltiorrhizae radix et Rhizoma* |
| C40 | isocryptotanshi-none | 54.98 | 0.39 | *Salviae miltiorrhizae radix et Rhizoma* |
| C41 | Isotanshinone II | 49.92 | 0.40 | *Salviae miltiorrhizae radix et Rhizoma* |
| C42 | miltionone Ⅰ | 49.68 | 0.32 | *Salviae miltiorrhizae radix et Rhizoma* |
| C43 | 1-methyl-8,9-dihydro-7H-naphtho[5,6-g]benzofuran-6,10,11-trione | 34.72 | 0.37 | *Salviae miltiorrhizae radix et Rhizoma* |
| C44 | prolithospermic acid | 64.37 | 0.31 | *Salviae miltiorrhizae radix et Rhizoma* |
| C45 | salvilenone Ⅰ | 32.43 | 0.23 | *Salviae miltiorrhizae radix et Rhizoma* |
| C46 | (6S)-6-(hydroxymethyl)-1,6-dimethyl-8,9-dihydro-7H-naphtho[8,7-g]benzofuran-10,11-dione | 65.26 | 0.45 | *Salviae miltiorrhizae radix et Rhizoma* |
| C47 | tanshinone Ⅵ | 45.64 | 0.30 | *Salviae miltiorrhizae radix et Rhizoma* |
| C48 | Salvigenin | 49.07 | 0.33 | *Salviae miltiorrhizae radix et Rhizoma* |
| C49 | Mono-O-Methylwightin | 103.11 | 0.40 | *Salviae miltiorrhizae radix et Rhizoma* |
| C50 | 2-(4-Hydroxy-3-Methoxyphenyl)-5-(3-Hydroxypropyl)-7-Methoxy-1-Benzofuran-3-Carbaldehyde | 62.78 | 0.40 | *Salviae miltiorrhizae radix et Rhizoma* |
| C51 | berberine | 36.86 | 0.78 | *Coptidis Rhizoma* |
| C52 | berberrubine | 35.74 | 0.73 | *Coptidis Rhizoma* |
| C53 | palmatine | 64.60 | 0.65 | *Coptidis Rhizoma* |
| C54 | epiberberine | 43.09 | 0.78 | *Coptidis Rhizoma* |
| C55 | (R)-Canadine | 55.37 | 0.77 | *Coptidis Rhizoma* |
| C56 | Berlambine | 36.68 | 0.82 | *Coptidis Rhizoma* |
| C57 | coptisine | 30.67 | 0.86 | *Coptidis Rhizoma* |
| C58 | Jatrorrhizine | 30.44 | 0.75 | *Coptidis Rhizoma* |
| C59 | Oxyberberine | 36.68 | 0.82 | *Coptidis Rhizoma* |
| C60 | Worenine | 45.83 | 0.87 | *Coptidis Rhizoma* |
| C61 | 5,2',5'-Trihydroxy-6,7,8-trimethoxyflavone | 37.49 | 0.43 | *Citrus sarcodactylis Fructus* |
| C62 | Diosmetin | 31.14 | 0.27 | *Citrus sarcodactylis Fructus* |
| C63 | Pectolinarigenin | 41.17 | 0.30 | *Cirsii japonici herba* |
| C64 | Dinatin | 30.97 | 0.27 | *Cirsii japonici herba* |
| C65 | DNOP | 40.59 | 0.40 | *Cirsii japonici herba* |
| C66 | ZINC03860434 | 43.59 | 0.35 | *Cirsii japonici herba* |
| C67 | Stigmasterol | 43.83 | 0.76 | *Notoginseng radix et Rhizoma、Cirsii japonici herba* |
